# Effect of familiarity and recollection during constrained retrieval on incidental encoding for new “foil” information

**DOI:** 10.3389/fpsyg.2022.957449

**Published:** 2022-09-14

**Authors:** Mingyang Yu, Can Cui, Yingjie Jiang

**Affiliations:** School of Psychology, Northeast Normal University, Changchun, China

**Keywords:** familiarity, recollection, constrained retrieval, foils, ERPs

## Abstract

Behavioral studies have demonstrated differences in the effect of constrained retrieval of semantic vs. non-semantic information on the encoding of foils. However, the impact of recognition on foils between semantic and non-semantic trials remains unclear. This study thus examines the roles of recognition—familiarity and recollection—in constrained retrieval for foils. We applied the event-related brain potentials (ERPs) data of new/old effects to elucidate the neural mechanisms underlying the “foil effect.” Participants encoded semantic and non-semantic tasks (Phase 1), were tested in a blocked memory task with new words presented as foils (Phase 2), and performed a surprise recognition task involving foils and completely new words (Phase 3). Behavioral results showed better recognition performance regarding reaction times and accuracy by hit and correct reject for semantic vs. non-semantic trials in Phase 2. Conversely, inferior recognition performance in reaction times and accuracy by hit and correct reject was noted for semantic vs. non-semantic foils in Phase 3. ERP results showed more positive Frontal N400 (FN400) for hit in non-semantic trials, more positive late positive component (LPC) for correct rejects in semantic trials in Phase 2, and more positive LPC for hits in both semantic and non-semantic trials only in Phase 3. Through dual-processing theory, we prove that different task types in constrained retrieval depend on different retrieval processes. Particularly, familiarity may be applied more often in non-semantic trials, and recollection in semantic trials. The difference in processes between semantic and non-semantic trials during constrained retrieval affects incidental encoding of foils.

## Introduction

The levels-of-processing (LoP) theory maintains that semantic processing tasks result in better memory storage compared to perceptual tasks (Craik and Tulving, 1975). According to the transfer-appropriate processing framework, higher retrieval success in semantic trials depends on deeper involvement of the cognitive operations engaged during encoding in the retrieval (Roediger et al., 2002; Hayama et al., 2008). This model suggests that memory retrieval entails re-implementing the neurocognitive processes involved during encoding; therefore, retrieval attempts also involve some degree of encoding (Buckner et al., 2001; Vogelsang et al., 2016).

In some studies, participants studied words in a semantic task block (pleasant/unpleasant judgment) and a non-semantic task block (letter judgment) (Phase 1). Next, a recognition memory test was administered (Phase 2) in which the studied and new words (“foils”) were intermixed. To participants’ surprise, there was a recognition test for semantic foils, non-semantic foils, and completely new words (Phase 3). Behaviorally, the recognition performance for “foil” words was significantly better for the semantic compared to the non-semantic condition (Jacoby et al., 2005; Danckert et al., 2011; Halamish et al., 2012; Messmer et al., 2020; Salhi and Bergstrom, 2020). Based on brain imaging, Vogelsang et al. (2016) revealed significant overlap in activities between Phases 1 and 2 for the semantic block in the left inferior frontal gyrus. Vogelsang et al. (2018) also observed that constrained retrieval of semantic information involved re-implementing semantic encoding operations mediated by alpha oscillations. It has thus been proposed that retrieval is strategically oriented toward the relevant processing mode to facilitate memory search (Jacoby et al., 2005; Halamish et al., 2012; Vogelsang et al., 2016, 2018.

Jacoby et al., 2005 state that recognition often involves source-constrained retrieval. The explanation for better incidental encoding of semantic foils compared to non-semantic foils is that the participants strategically constrain their retrieval to match a semantic processing mode while attempting to recognize semantic probe words, and a non-semantic processing mode while recognizing non-semantic information (Marsh et al., 2009; Alban and Kelley, 2012; Halamish et al., 2012). This viewpoint is similar to the concept of “retrieval orientation” in Rugg and Wilding (2000), which refers to the type of processing that participants engage in when they are prompted with a retrieval cue to increase the likelihood of retrieval success. Rugg and Birch, 2000 also indicate that the depth of the study processing evokes a different old/new effect. The “old/new effect” has been interpreted as evidence that memory retrieval engages a range of naturally and functionally distinct processes. It refers to the phenomenon in which event-related brain potentials (ERPs) elicited by a hit (correctly identifying old items as “old”) have more positive-going amplitudes compared to a correct rejection (correctly identifying new items as “new”) (Rugg and Curran, 2007; Hayama et al., 2008; Halamish et al., 2012).

Rugg and Birch, 2000 analyzed the ERP differences between semantic and non-semantic conditions in the recognition phase. The ERPs elicited by new words in the block following the non-semantic study task exhibited more positive-going waveforms. The late old/new effects were only evoked in the semantic condition, whereas the early old/new effects were evoked for both the semantic and non-semantic studied words. Together, these findings indicate that the depth of the study processing influences the different neural activities associated with memory search operations as well as the processing of retrieved information.

Furthermore, the dual-process theory of recognition memory states that recognition decisions can be based on either recollection or familiarity (Rugg and Curran, 2007). However, familiarity-based recognition does not provide qualitative information about the study episode. Meanwhile, recollection is a more effort-intensive process that gives rise to consciously accessible information on prior and later occurrences of the test item (Yonelinas, 2001). Frontal N400 (FN400, also called the early old/new effect) has been associated with the familiarity process, and the late positive component (LPC, also called late old/new effect) with recollection (Allan et al., 1998; Friedman and Johnson, 2000; Rugg and Curran, 2007). According to this view, the late old/new effect in the semantic condition in Rugg and Birch (2000)) reflects recollection-based recognition, while the early old/new effect in both the semantic and non-semantic conditions reflects familiarity-based recognition (Rugg and Curran, 2007). This suggests that both familiarity and recollection are required in a semantic recognition task, while only familiarity is required in a non-semantic task.

Buckner et al. (2001) found that encoding occurs even during retrieval tasks, and the foil effect provides evidence for the difference in foil recognition performances between the semantic and non-semantic conditions (Jacoby et al., 2005; Marsh et al., 2009; Danckert et al., 2011; Zawadzka et al., 2017; Vogelsang et al., 2018; Salhi and Bergstrom, 2020). It implies that participants will encode all words during the recognition test, irrespective of whether they are old or foils. Furthermore, the difference in memory performance between semantic and non-semantic foils arises from the strategic retrieval orientation (Jacoby et al., 2005; Danckert et al., 2011; Salhi and Bergstrom, 2020). Thus, the roles of familiarity and recollection during incidental foil encoding differ in semantic vs. non-semantic conditions (Rugg and Birch, 2000; Rugg and Curran, 2007). Unfortunately, the ERP results of this LoP effect have not been incorporated into the foil effect explanation. Instead, the literature has tended to focus on behavioral (Alban and Kelley, 2012; Zawadzka et al., 2017; Salhi and Bergstrom, 2020) and brain imaging studies (Vogelsang et al., 2016, 2018; Messmer et al., 2020).

Therefore, in the current study, we compared both the foil effect and the old/new effect in the memory-for-foils paradigm directly, to investigate the effect of the retrieval strategy on the encoding of new words that were added as foils in Phase 2. We assumed that familiarity and recollection played different roles during the incidental encoding for “foils” in semantic and non-semantic trials when constrained retrieval is accrued. In particular, we predicted that, in line with prior findings (Jacoby et al., 2005; Marsh et al., 2009), final recognition would be enhanced for foils previously shown in the semantic condition compared to non-semantic foils in behavioral terms. We also expected that the semantic condition would evoke both FN400 and LPC, while the non-semantic condition would only evoke FN400 in Phase 2 and produce the larger amplitude, in line with prior evidence that the depth of study processing modulates retrieval orientation (Rugg and Birch, 2000; Rugg and Curran, 2007). Based on dual-process theory, we predicted a smaller difference between semantic and non-semantic foils in Phase 3. Specifically, both semantic and non-semantic foils are incidentally encoded by the participants in Phase 2. Hence, the available information is limited in the final recognition test, and the participants will evoke LPC when they try to recollection. Furthermore, the semantic condition produces the more positive amplitude.

## Materials and methods

### Participants

The participants were 21 students (9 men) aged 18–26 years (*M* = 20.24 years, SD = 1.85). All had normal or corrected-to-normal visual acuity and did not have any history of neurological or mental disease. The number of participants per group was similar to that in Vogelsang et al.’s (2016) experiment (*N* = 22), which used a similar paradigm. A sensitivity analysis using G* Power 3.1 (Faul et al., 2009) revealed that, assuming a power of 0.80 with our sample size (*N* = 21), the experiment was sufficiently sensitive to detect an effect size of 0.64 for paired samples *t*-tests. The Research Ethics Committee of the Northeast Normal University of China approved this study. Participants provided written informed consent, per the Declaration of Helsinki.

### Materials

The stimuli consisted of 432 Chinese words from the *Modern Chinese Frequency Dictionary.* These words were split into six lists according to familiarity (*M* = 5.25, SD = 0.72) and frequency (*M* = 1.86, SD = 2.50). The assignment of the lists was balanced according to the experimental conditions of the participants.

### Procedure

**Figure 1:**
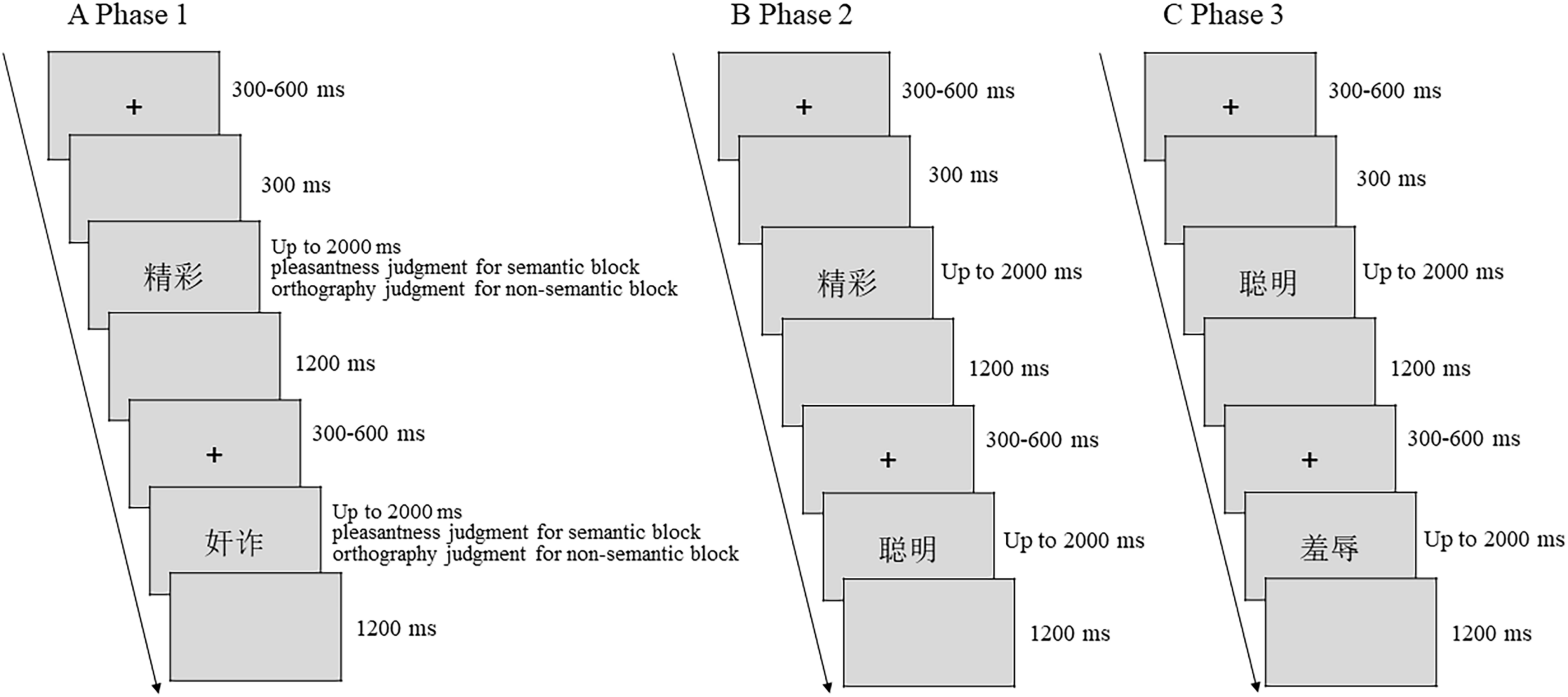
Memory-for-foils paradigm of the experiment. All variables were manipulated within subjects.

Participants were fitted with an electroencephalogram (EEG) cap and seated in a sound-and light-attenuated room. During Phase 1, participants made semantic judgments (“Is this word pleasant?”) for 72 words and non-semantic judgments (“Does this word have left–right construction?”) for 72 words in two different study blocks. An instruction was presented at the beginning of each block to remind participants about the presence of a semantic or non-semantic block. Trials started with a randomly jittered 300–600 ms fixation cross, followed by a blank screen for 300 ms; finally, the stimulus was presented at the center of the screen for 2,000 ms. During this time, participants were asked to complete their semantic judgments (semantic block) or non-semantic judgments (non-semantic block) by pressing the “F” or “J” key on the keyboard. If participants did not respond within the duration of stimulus presentation, the stimulus was removed from the screen. The next trial started after a 1,200 ms blank screen (Figure 1, A Phase 1).

In Phase 2, the participants were given an old/new recognition test. In the semantic block, 72 old words from the semantic study phase were intermixed with 72 new words (semantic foils). In the non-semantic block, 72 old words from the non-semantic study phase were intermixed with 72 new words (non-semantic foils). Each test trial started with a randomly jittered 300–600 ms fixation cross, followed by a blank screen of 300 ms, and then a stimulus presented at the center of the screen for 2,000 ms. During this time, participants were asked to determine whether the word was “Old” (studied word) or “New” (unstudied word) by pressing the “F” or “J” keys, respectively, on the keyboard. If participants did not respond within the duration of stimulus presentation, the stimulus was removed from the screen. The next test trial started after a 1,200 ms blank screen. Participants were told in advance that the old items were from the Phase 1 semantic or non-semantic blocks (Figure 1, B Phase 2).

In Phase 3, we administered a surprise source memory test. Participants were asked to distinguish between the 72 semantic foils, 72 non-semantic foils, and 144 entirely new words. Each trial in the final foil recognition test started with a randomly jittered 300–600 ms fixation cross, followed by a blank screen of 300 ms, and then a stimulus presented at the center of the screen for 2,000 ms. During this time, participants were asked to determine whether a word was old or new by pressing the “F” and “J” keys on the keyboard, respectively. If participants did not respond within the duration of stimulus presentation, the stimulus was removed from the screen. The next foil test trial started after a 1,200 ms blank screen (Figure 1, C Phase 3).

### Data recording and analyses

#### Behavioral data

For the data of Phase 2, the mean proportions of hits of semantic/non-semantic words and correct rejections of semantic/non-semantic foils were calculated. For the data of Phase 3, the mean proportions of hits of semantic/non-semantic foils were calculated. Finally, we analyzed the response accuracy and response time (RT) for both phrases using paired samples *t-*tests with task types (semantic, non-semantic) as the within-subject factor.

#### Event-related potentials

We recorded brain electrophysiological activity using the Neuroscan system according to the extended international 10–20 system using 62 Ag/AgCI electrodes positioned in an elastic nylon cap, with the reference on the left mastoid. We positioned the electrodes above and below the left eye, and on the left and right canthi of the eyes to record the vertical and horizontal electrooculograms, respectively. The impedance of all electrodes was maintained at below 10 KΩ. The EEG and electrooculogram were amplified using a 0.05–100 Hz band-pass and continuously sampled at 1,000 Hz.

Off-line analyses were performed in MATLAB using the EEGLAB (Delorme and Makeig, 2004) and ERPLAB toolbox (Lopez-Calderon and Luck, 2014). The EEGs were filtered using IIP-Butterworth filters with 30 Hz low-pass and 0.1 Hz high-pass filters (Luck, 2014). After independent component analysis for ocular correction, we supplemented the artifact correction process with artifact rejection to eliminate trials with clear artifactual voltage deflections or when peak-to-peak voltage within the EEG epoch exceeded 300 μV in any 200 ms window in any channel (Lopez-Calderon and Luck, 2014; Bacigalupo and Luck, 2018). We segmented the ERPs for all trials into 1,000 ms epochs surrounding the stimulus onset and corrected the baseline to account for the 200 ms pre-stimulus epoch.

Based on the grant-averaged ERPs of different waveforms, our ERP analysis strategy was similar to that of previous studies that analyzed the FN400 and LPC components (Duzel et al., 1997; Vilberg et al., 2006; Rugg and Curran, 2007). We analyzed the ERP data from F3, F4, P3, and P4. We conducted separate analyses for the 350–450 and 700–800 ms time windows, corresponding to the FN400 and LPC epochs, respectively. In Phase 2, a three-factor repeated-measures analysis of variance (ANOVA) with 2 (task type: semantic, non-semantic) × 2 (response: hit, correct reject) × 4 (electrode: F3, F4, P3, and P4) as within-subject factors was performed on mean amplitudes for the FN400 and LPC epochs. In Phase 3, a three-factor repeated-measures ANOVA with 2 (task type: semantic, non-semantic) × 3 (response: semantic hit, non-semantic hit, correct reject) × 4 (electrode: F3, F4, P3, and P4) as within-subject factors was performed. Greenhouse–Geisser correction was performed when the assumption of sphericity was violated for a particular sample. Holm corrections were used to adjust for multiple comparisons.

## Results

### Reaction time and accuracy

In Phase 2, hits were higher for semantic trials than for non-semantic trials [*M* = 0.77 ± 0.03 vs. *M* = 0.70 ± 0.02, respectively; *t* (20) = 2.713, *p* = 0.013, 95% CI (0.02, 0.12), Cohen’s *d* = 0.60]. Correct rejection of foils was higher for semantic trials than for non-semantic trials [*M* = 0.68 ± 0.02 vs. *M* = 0.55 ± 0.03, respectively; *t* (20) = 5.71, *p* < 0.001, 95% CI (0.08, 0.17), Cohen’s d = 1.25]. The RTs of hits were shorter for semantic trials than for non-semantic trials [*M* = 850.86 ± 20.45 vs. *M* = 891.20 ± 31.21, respectively; *t* (20) = 2.18, *p* = 0.042, 95% CI (1.70, 78.98), Cohen’s *d* = 0.48]. There was no significant difference in the RTs of correct rejections between semantic and non-semantic trials [*t* (20) = 1.50, *p* > 0.15] (Table 1).

**Table 1:** Performance during Phase 2 and Phase 3 in the semantic and non-semantic conditions.

	Recognition accuracy				Reaction time (ms)			
	Old words		New words		Old words		New words	
	Semantic	Non-semantic	Semantic	Non-semantic	Semantic	Non-semantic	Semantic	Non-semantic
Phase 2	0.77 (0.03)	0.70 (0.02)	0.68 (0.02)	0.55 (0.03)	850.86 (20.45)	891.20 (31.21)	919.49 (26.91)	942.19 (27.44)
Phase 3	0.32 (0.03)	0.44 (0.02)	0.51 (0.03)		1078.54 (40.96)	1041.60 (37.22)	987.30 (27.83)	

Note: Values are Mean (SD).

In Phase 3, hits were lower for semantic foils than for non-semantic foils (*M* = 0.32 ± 0.03 vs. *M* = 0.44 ± 0.02). The RTs of hits were marginally longer for semantic foils than for non-semantic foils [*M* = 1078.54 ± 40.96 vs. *M* = 1041.60 ± 37.22, respectively; *t* (20) = 1.89, *p* = 0.074, 95% CI (−3.94, 77.82), Cohen’s *d* = 0.48] (Table 1).

### Event-related potentials

In Phase 2, during the FN400 (350–450 ms) epoch, we found a main effect of response [*F*(1,20) = 6.29, *p* = 0.02, *η_p_*^2^ = 0.24], with post-hoc multiple comparisons revealing that hits were significantly more positive than correct rejects (*p* = 0.02). A marginally significant main effect of task type was also observed [*F*(1,20) = 3.74, *p* = 0.06, *η_p_*^2^ = 0.16], with post-hoc multiple comparisons revealing that the non-semantic condition was marginally significantly more negative than the semantic condition (*p* = 0.06). A significant interaction effect between the task type and response was observed [*F*(2,20) = 5.98, *p* = 0.02, *η_p_*^2^ = 0.23]. A simple effects analysis showed that FN400 was more negative for correct rejects of foils than for hits of old words in non-semantic trials (*p* = 0.002) and that there was no FN400 effect in semantic trials; non-semantic trials were significantly more negative than semantic trials in terms of hits for old words (*p* = 0.01). This result indicates that the recognition process in non-semantic trials is more dependent on familiarity than in semantic trials. No other significant main or interaction effects were observed.

**Figure 2:**
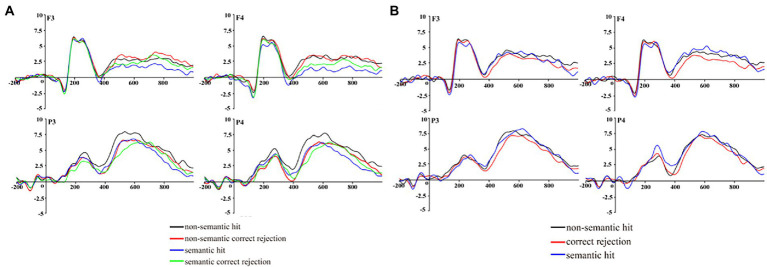
Grand averaged event-related potentials for Phase 2 **(A)** and Phase 3 **(B)**.

During the LPC (700–800 ms) epoch, we found a main effect of task type [*F*(1,20) = 6.57, *p* = 0.02, *η_p_*^2^ = 0.25] and a significant interaction effect between the task type and response [*F*(2,20) = 7.59, *p* = 0.01, *η_p_*^2^ = 0.28]. A simple effects analysis showed that LPC was more positive for correct rejects of foils than for hits of old words in semantic trials (*p* = 0.006), but not so for the non-semantic trials (*p* = 0.35); non-semantic trials were significantly more positive than semantic trials in terms of hits for old words (*p* = 0.001). This result indicates that the recognition process in semantic trials is more dependent on recollection than in non-semantic trials. No other significant main or interaction effects were observed (*ps >* 0.1) (Figure 2A).

In Phase 3, during the FN400 epoch, no significant main or interaction effects were observed (*ps >* 0.1). During the LPC epoch, we found a main effect of task type [*F*(2,40) = 3.03, *p* = 0.04, *η_p_*^2^ = 0.14]. Post-hoc multiple comparisons revealed that non-semantic hits for foils were significantly more positive than were correct rejects for new words (*p* = 0.03); semantic hits for foils were significantly more positive than were correct rejects for new words (*p* = 0.04). No significant main effect for electrode [*F*(3,60) = 0.93, *p >* 0.1] or interaction effects between task type and electrode [*F*(6,120) = 0.78, *p >* 0.1] were observed (Figure 2B).

## Discussion

The aim of this study was to investigate the effect of the retrieval strategy involved in the successful encoding of new “foil” information presented during a recognition test when participants engaged in a semantic vs. non-semantic task. In doing so, we demonstrate the influence of the retrieval strategy in different tasks on foils encoding in recognition tests, and further discuss differences in the foil effect between Chinese and English character materials.

Our behavioral results showed that the recognition performance in the semantic condition was better than in the non-semantic condition in Phase 2, thus supporting the results from earlier studies by demonstrating the typical LoP effect (Rugg and Wilding, 2000; Roediger et al., 2002). Notably, the foil effect refers to semantic foils, which were remembered significantly more accurately than non-semantic foils in Phase 3 (Jacoby et al., 2005; Marsh et al., 2009; Salhi and Bergstrom, 2020). However, interestingly, we found higher accuracy and shorter RTs for non-semantic foils. To investigate the influence of the recognition process on foils encoding, we focused on the analysis of old/new effects in the recognition test, thereby providing a new perspective for clarifying the mechanism underlying the foil effect. In Phase 2, there was significant FN400 in the non-semantic condition and LPC in the semantic condition. To some extent, this finding is similar to previous results (Rugg and Wilding, 2000; Rugg and Curran, 2007). There was an LPC effect in both the semantic and non-semantic foils in Phase 3.

Clearly, the behavioral results in Phase 3 are significantly different from the foil effect. By comparing previous studies and our research, it is evident that both semantic and non-semantic tasks are used to control processing depth. However, whereas English was used as the experimental material in previous studies (Jacoby et al., 2005; Halamish et al., 2012; Vogelsang et al., 2016, 2018), this study used Chinese; the orthographic characteristics of words in Chinese and English affect memory performance (Phase 3) for foils.

English is different from Chinese in terms of the representations and mappings between orthography, phonology, and semantics (Booth et al., 2006). English is an alphabetic language whereas Chinese is a logographic language, with less systematic information on phonology (Zhu et al., 2014). In English, the structure of a word is fixed by the order of the letters from left to right, with most of the letters having one pronunciation. The composition of Chinese characters, which uses radicals, does not follow one-to-one pronunciation rules (Booth et al., 2006; Zhu et al., 2014; Tian et al., 2020). Thus, compared to English, Chinese has a different orthographic system that has more clues to semantics (Booth et al., 2006; Tian et al., 2020; Wu et al., 2020). Chinese characters encode meaning by including a semantic radical (Booth et al., 2006; Zhu et al., 2014; Liu et al., 2020). Therefore, participants judged the orthographic characteristic of the words in the non-semantic study task, and there was a by-product of semantic information in Phase 1. Therefore, the recognition test in non-semantic conditions mainly relies on the familiarity of glyph features. This causes the participants to pay attention to the glyph features of all the test words, thereby enhancing their memory of new words. In the semantic task, the participants generated rich details during pleasure judgment, making the judgments based on one or more specific pieces of detailed information during recollection. However, this causes the participants to ignore the processing of other information related to the test words and weakens their memory of new words. Therefore, in Phase 2, the semantic and non-semantic conditions were driven by recollection and familiarity, respectively. This difference led to differences in the encoding level of new words under the two conditions. The difference in orthography between Chinese and English resulted in better memory performance in relation to non-semantic foils in the final recognition test.

In Phase 2, the ERP effect differed in semantic vs. non-semantic trials. Specifically, we observed the FN400 component on non-semantic trials, which is associated with familiarity, and the LPC component on semantic trials, which is associated with recollection, in line with previous opinion. This result indicates that the non-semantic test block mainly depends on familiarity, whereas the semantic test block mainly depends on recollection in constrained retrieval (Friedman and Johnson, 2000; Rugg and Birch, 2000; Rugg and Wilding, 2000). However, there was an LPC effect in both the semantic and non-semantic foils in Phase 3, in line with our hypothesis. This suggests that the participants engage in recollection during the final recognition test, which can be inferred from previous literature. In the memory-for-foils paradigm, participants did not know the existence of Phase 3 in advance (Jacoby et al., 2005; Vogelsang et al., 2016; Salhi and Bergstrom, 2020), so they did not consciously memorize test words, especially foils, in Phase 2. Therefore, when the final surprise recognition test was administered, participants tried to recollect relevant details to improve their memory performance.

Of note, the LPC was more positive for correct rejects than for hits during semantic trials in Phase 2. Previous recognition memory research has suggested that the old/new effect is associated with decision accuracy and participants’ confidence about familiarity and recollection (Finnigan et al., 2002; Gao et al., 2019). According to this view, the reversed LPC suggests that participants were less confident about old words than about new words in the semantic test block.

In the non-semantic task, the participants formed perceptual memories of word shapes and radicals, and the familiarity thus generated helped word recognition. The participants compared the memorized information with the test words in this process. Consequently, they incidentally embedded similar perceptual information in non-semantic foils. In the semantic task, the participants generated accurate semantic information for test words, which helped quickly distinguish between words. Therefore, semantic foils had less embedded information. The difference in the information embedded in foils led to better incidental memory for non-semantic foils.

## Conclusion

The present study used FN400 and LPC to delineate the influences of familiarity from those of recollection on incidental encoding for new foils during constrained retrieval. Behavioral results indicated that semantic trials that performed better in Phase 2 performed worse in Phase 3, while non-semantic trials that performed worse in Phase 2 performed better in Phase 3. The ERP results indicated that non-semantic and semantic trials evoked FN400 and LPC, respectively, in Phase 2, but both evoked LPC in Phase 3. This study thereby demonstrated that constrained retrieval is associated with familiarity and recollection during non-semantic and semantic trials, respectively. Different retrieval strategies affect incidental encoding for new words as foils during semantic and non-semantic trials, and the difference may be influenced by the perceptual information involved in the study materials.

## Data availability statement

The raw data supporting the conclusions of this article will be made available by the authors, without undue reservation.

## Ethics statement

The studies involving human participants were reviewed and approved by the Research Ethics Committee of the Northeast Normal University of China. The patients/participants provided their written informed consent to participate in this study.

## Author contributions

MY and YJ contributed to the conception and design of the study. MY organized the database and wrote the first draft of the manuscript. MY and CC performed the statistical analysis. All authors contributed to the article and approved the submitted version.

## Funding

This study was supported by the National Social Science Foundation of China (19BSH113).

## Conflict of interest

The authors declare that the research was conducted in the absence of any commercial or financial relationships that could be construed as a potential conflict of interest.

## Publisher’s note

All claims expressed in this article are solely those of the authors and do not necessarily represent those of their affiliated organizations, or those of the publisher, the editors and the reviewers. Any product that may be evaluated in this article, or claim that may be made by its manufacturer, is not guaranteed or endorsed by the publisher.

## Author Disclaimer

This manuscript has not been published or presented elsewhere in part or in entirety and is not under consideration by another journal. All study participants provided informed consent, and the study design was approved by the appropriate Ethics Review Board.

## Abbreviations

ERP: event-related brain potential
EEG: electroencephalogram
FN400: frontal N400
LPC: late positive component
LoP: levels-of-processing
RT: response time.
